# Determining the effect of collective efficacy and teacher autonomy on positive organizational behavior in physical education and sports teachers using structural equation modeling

**DOI:** 10.3389/fpsyg.2026.1773257

**Published:** 2026-04-08

**Authors:** Nuri Berk Gungor, Oruc Ali Ugur, Tebessum Ayyildiz Durhan, Serkan Kurtipek, Alptug Soyer, Selahattin Akpinar

**Affiliations:** 1 Faculty of Sport Sciences, Balikesir University, Balikesir, Türkiye; 2 Faculty of Sport Sciences, Karamanoglu Mehmetbey University, Karaman, Türkiye; 3 Faculty of Sport Sciences, Gazi University, Ankara, Türkiye; 4 Faculty of Sport Sciences, Nigde Omer Halisdemir University, Nigde, Türkiye; 5 Faculty of Sport Sciences, Duzce University, Duzce, Türkiye

**Keywords:** autonomy, collective efficacy, organizational behavior, physical education, teacher

## Abstract

**Background:**

One of the important employees in the field of sports is physical education and sports teachers. The responsibility of the relevant workforce to direct students in young age groups to sports branches in formal education areas and to develop them is remarkable. For this reason, shedding light on the characteristics of physical education and sports teachers will benefit the development of the field of sports.

**Methods:**

Within the scope of the research conducted to determine the effect of collective efficacy and autonomy of physical education and sports teachers on positive organizational behavior with structural equation modeling, 250 physical education and sports teachers were included in the study. In this context, personal information form and Teacher Autonomy, Collective Teacher Efficacy and Positive Organizational Behavior Scales were used in the study. The theoretical model created in the analysis of the data was tested with structural equation modeling. Also, Independent Sample *T*-Test, Pearson Correlation Test and descriptive statistics were utilized.

**Results:**

The findings show that the participants have high levels of teacher autonomy, and average levels of collective efficacy and positive organizational behavior. It was determined that the measurement tools did not differ according to the gender variable, and there was a positive and moderate relationship between the participants’ teacher autonomy and collective efficacy. In addition, it was determined that there was a positive and moderate relationship between the participants’ teacher autonomy and their positive organizational behavior levels. The findings of the structural equation model indicate that there is a statistically significant effect between teacher autonomy and collective efficacy and positive organizational behavior.

**Conclusion:**

As a result, it has been determined that the related parameters have a relationship and effect with each other and the model formed in line with the hypotheses supports this.

## Introduction

It is known that physical education and sport teachers play an important role in the academic and social–emotional development of students. In order to fulfill this role effectively, it is important for teachers to have the necessary knowledge, skills and attitudes, i.e., high collective efficacy. In addition, teachers’ autonomy in planning and implementing their work increases their motivation and job satisfaction and contributes to more positive organizational behaviors.

Physical education and sport teachers are teachers who plan, implement and evaluate physical education and sport lessons. The aim of these courses is to support the physical, mental and social–emotional development of students. Physical education and sport teachers should have various knowledge, skills and attitudes to achieve this goal. Among these knowledge, skills and attitudes, collective efficacy is one of the characteristics that are gaining importance in our age. Collective efficacy is a phenomenon formed by the combination of knowledge, skills and attitudes necessary for individuals in a group to achieve a common goal (Bandura, 1997). Collective efficacy emerges when group members focus on a common goal and work together to achieve this goal (Dansereau, 1997). Group members depend on each other to achieve the common goal. Therefore, it is important for group members to communicate and cooperate with each other (Johnson and Johnson, 1989). Each group member brings different knowledge, skills and attitudes to the group. The combination of these different skills creates collective efficacy (Katzenbach and Smith, 1993).

By providing social support to each other, group members help each other overcome difficulties and achieve their goals (Gronn, 2000). With collective efficacy, group members show higher performance by working together to achieve a common goal (Salas et al., 2005), show more productivity and do more work in less time (Hackman, 1987), produce more innovative ideas by sharing different perspectives and skills (West, 2002), solve problems easier and faster by working together (Wageman, 1995), and become more motivated by receiving support and encouragement from each other (Hoyle, 1999). In this context, it is important to evaluate the positive characteristics of physical education teachers such as collective efficacy. Focusing on a common goal and working together to achieve this goal through increasing collective efficacy contributes to individuals and overall functioning (Dansereau, 1997). It is important for group members to share their knowledge and skills and to support each other (Johnson and Johnson, 1989), to have different skills (Katzenbach and Smith, 1993) and to help each other overcome difficulties by providing social support (Gronn, 2000). Collective efficacy among physical education and sport teachers contributes to more effective planning and implementation of lessons by increasing teachers’ cooperation and coordination with each other.

Autonomy is the individual’s freedom to plan and implement his/her own work. In other words, autonomy is the freedom to control one’s own behavior and make one’s own decisions (Deci and Ryan, 2000). This concept has an important place in many fields such as psychology and philosophy. Autonomous individuals are generally characterized by a lower dependence on external instructions or direct control from others (Ryan and Deci, 2008). Autonomous individuals have the ability to manage their own lives and set their own goals (Gagne and Deci, 2005) and are ready to take responsibility for their own choices (Ryan and Deci, 2001).

Autonomy has a significant impact on an individual’s motivation, well-being and psychological health. Individuals who can make their own choices and set their own goals are more motivated to achieve these goals (Deci and Ryan, 2000). Autonomous individuals tend to report higher levels of well-being and life satisfaction, possibly due to their perceived sense of control (Ryan and Deci, 2002) and tend to have lower levels of stress and anxiety (Gagne and Deci, 2005). Giving other individuals the freedom to control their own behavior and make their own decisions is one of the most important ways to increase autonomy (Ryan and Deci, 2008). Giving responsibility to individuals helps them to take control of their own lives and develop their autonomy (Gagne and Deci, 2005). Supporting individuals to help them achieve their own goals contributes to the development of their autonomy (Ryan and Deci, 2002). For physical education and sport teachers, autonomy refers to the freedom to plan and implement their lessons in their own way and with their own content. Autonomy increases teachers’ motivation and job satisfaction and contributes to more positive organizational behavior.

Positive organizational behavior refers to the behaviors that employees demonstrate at the workplace and contribute to the goals of the organization. These behaviors include concepts such as job commitment, job satisfaction, motivation, productivity, innovation and teamwork. It is a management approach that focuses on increasing the well-being and productivity of employees. This approach aims to create a more positive and motivating environment in the workplace by focusing on employees’ strengths and potentials (Cameron and Spreitzer, 2012). It aims to help employees develop and utilize their strengths instead of trying to correct their weaknesses (Luthans and Avolio, 2003).

Positive organizational behavior outcomes try to create an environment that will enable employees to be more committed to their jobs and organizations (Kramer and Dutton, 2001). It also aims to make them happy and healthy at work (Diener and Biswas-Diener, 2008). It aims to contribute to the overall success of the organization by making employees more motivated and productive (Hackman and Oldham, 1976). Giving feedback to employees about their strengths and areas that they need to improve (Avolio and Luthans, 2006), setting achievable and motivating goals with employees (Locke and Latham, 1990), appreciating and rewarding employees’ achievements and contributions (Deci and Ryan, 2000), building strong and supportive relationships among employees and between managers and employees, and designing jobs in a way that makes employees feel that their work is meaningful and important (Hackman and Oldham, 1976) are extremely important. Positive organizational behavior has been associated with higher levels of employee engagement, well-being, productivity, and creativity, and may contribute to lower turnover rates (Kramer and Dutton, 2001), well-being (Diener and Biswas-Diener, 2008) and productivity (Hackman and Oldham, 1976), decreases turnover rates (Luthans and Avolio, 2003), and increases creativity and innovation (Amabile, 1996). Therefore, positive organizational behavior outcomes are an effective parameter for employee productivity.

In the light of all this information, it is important to describe the characteristics of collective efficacy, autonomy and positive organizational behavior, which are seen as important for the educational understanding of our age, in the focus of physical education and sports teachers. At this point, it is foreseen to contribute to innovative education models and organizational quality and to increase the studies for the goal of improvement in school climate. To the best of our knowledge, studies directly examining the proposed model are limited, which highlights the potential contribution of the present research. In order to examine the relationships between more than one variable, the current research was put forward to determine the relationships between both observable and unobservable variables with the structural equation model. In this direction, the aim of the research is to examine the effect of collective efficacy and autonomy of physical education and sports teachers on positive organizational behavior.

## Materials and methods

### Study design

In this study, which aims to determine the effect of teacher autonomy and collective efficacy on positive organizational behavior, the relational survey model was applied. Relational survey model; “is used to determine the relationship between two or more variables and to obtain clues about cause and effect (Karasar, 2007).” The theoretical model established as a result of the related literature review was tested with the structural equation model. The structural equation model is “a combination of regression and factor analysis and is a theoretical structure represented by latent and observed variables (Byrne, 2013).” The hypotheses used in the model are presented below;

*H_1_*: There is a statistically significant relationship between teacher autonomy (TA) and positive organizational behavior (POB).

*H_2_*: There is a statistically significant relationship between collective efficacy (CTE) and positive organizational behavior (POB).

### Study sample

In line with the research objective, the study was conducted with officially employed physical education and sports teachers in the central districts of Ankara. In addition, the research was limited to Ankara province. Data collection took place between February and June 2025. A disproportionate cluster sampling method was used in the sample selection process. The most significant benefit of cluster sampling is that it reduces costs by preventing the researcher from spreading over a large physical area and provides advantages in control capabilities. First, a list of schools in Ankara where 1,819 physical education and sports teachers work was obtained. The sample size was narrowed down according to the principle of impartiality and inclusion. Data collection was then completed with 325 volunteers from among the physical education and sports teachers currently working at the selected schools. This research was conducted in schools located in the center of Ankara. Since the number of teachers in each school varies, visits were made to the schools until a sufficient number of participants was reached, and data collection continued with participants who volunteered to participate in the research. Because the teachers were assigned to these schools through the same process, there were no differences in theoretical qualifications among the teachers. Therefore, schools with a higher number of teachers were prioritized. No exclusion criteria were applied among physical education and sports teachers in the participant selection process.

This study was designed in accordance with the fundamental ethical principles of the Declaration of Helsinki, and all procedures, including the protection of participant rights, the assurance of voluntary participation, and the confidentiality of all collected data, were conducted within the standards prescribed by the authorized ethical commissions.

The study group of the research consisted of 250 physical education and sports teachers, 109 (43.6%) female and 141 (56.4%) male, who were working in Ankara in 2025. Of the participants, 130 (52%) were working in secondary school and 120 (48%) in high school. In addition, 23 (10.4%) of the participants have 1–5 years of service, 41 (16.4%) 6–10 years, 34 (13.6%) 11–15 years, 50 (20%) 16–20 years and 99 (39.6%) 21 years or more. In addition, when the educational level of the participants was examined, 214 (85.6%) had bachelor’s degrees, 32 (12.8%) had master’s degrees and 4 (1.6%) had doctoral degrees. The principle of easy accessibility was taken into consideration when selecting the study group.

### Data collection tools

In the study, personal information form and Teacher Autonomy, Collective Teacher Efficacy and Positive Organizational Behavior Scales were used.

### Teacher Autonomy Scale

The scale was developed by Çolak and Altınkurt (2017) and consists of 17 items and 4 sub-dimensions. The sub-dimensions are named as “teaching process autonomy,” “curriculum autonomy,” “professional development autonomy” and “professional communication autonomy.” The items are scored based on a 5-point, Likert-type scale with increased scores signifying increases in teachers’ autonomy behaviors. Cronbach’s *α* internal consistency coefficients were observed to be between 0.78 and 0.89 (Çolak and Altınkurt, 2017). The internal consistency coefficients obtained from the scale are 0.75, 0.80, 0.87, 0.85 for the sub-dimensions, respectively. The internal consistency coefficient for the whole scale was found to be 0.86.

### Collective Teacher Efficacy Scale

The scale developed by Tschannen-Moran and Barr (2004) was adapted into Turkish by Erdoğan and Dönmez (2015). It consists of 12 items and 2 dimensions in total. The sub-dimensions are named as “student discipline” and “teaching strategies.” The increase in the total score obtained from the scale is interpreted as an increase in the related trait. The internal consistency coefficients obtained from the data set were 0.89 and 0.84 for the sub-dimensions and 0.93 for the whole scale, respectively.

### Positive Organizational Behavior Scale

Positive Organizational Behavior Scale was developed by Luthans et al. (2007). It consists of 24 items and 4 sub-dimensions. The sub-dimensions are; “optimism,” “psychological resilience,” “hope” and “self-efficacy.” It can be stated that the total score obtained from the scale increases the participants’ positive organizational behavior. While the internal consistency coefficient determined for the whole scale is 0.92, it is 0.84, 0.68, 0.75 and 0.84 for the sub-dimensions, respectively.

### Confirmatory factor analysis of the measurement instruments used in the study

Considering Table 1, it can be seen that the goodness-of-fit values related to the confirmatory factor analysis results obtained from the measurement instruments used in the study fall within the excellent value range and acceptable range (Tabachnick and Fidell, 2013). In addition to the relevant values, the AVE value for convergent validity was determined as TA: 0.67, CTE: 0.78, POB: 0.83; and the CR value as TA: 0.91, CTE: 0.81, POB: 0.96. Considering the literature, it is seen that an AVE value below 0.50 is acceptable if the CR value is above 0.70. Therefore, it is seen that the conditions for convergent validity are met. As a result of this, it can be stated that the factor structures of the measurement instruments have been validated and the prerequisite for using structural equation modeling has been met.

**Table 1:** Confirmatory factor analysis of the measurement instruments used in the study.

Model fit index	Excellent value range	Acceptable range	TA	CTE	POB
*χ*^2^/df	0 < *χ*^2^/df < 2	2 < *χ*^2^/df < 5	1.91	1.52	2.02
RMSEA	0.00 < RMSEA < 0.05	0.05 < RMSEA < 0.10	0.06	0.05	0.07
PGFI	0.95 < PGFI < 1.00	0.50 < PGFI < 0.95	0.73	0.94	0.72
PNFI	0.95 < PNFI < 1.00	0.50 < PNFI < 0.95	0.78	0.71	0.77
GFI	0.90 < GFI < 1.00	0.85 < GFI < 0.90	0.89	0.91	0.86
AGFI	0.90 < AGFI < 1.00	0.85 < AGFI < 0.90	0.87	0.88	0.85
CFI	0.95 < CFI < 1.00	0.90 < CFI < 0.95	0.91	0.98	0.90

*χ*^2^/df, chi-square divided by degrees of freedom; RMSEA, root mean square error of approximation; PGFI, parsimony goodness-of-fit index; PNFI, parsimony normed fit index; GFI, goodness-of-fit index; AGFI, adjusted goodness-of-fit index; CFI, comparative fit index.

### Statistical analysis

A power analysis was conducted to ensure generalizability of the research results. It was calculated that a minimum sample size of 317 was required for a known population (*n*: 1819), with a 95% confidence interval and a 5% margin of error. Initially, 325 physical education and sports teachers were included in the study due to difficulties with the consent procedures. Analysis of the data revealed that 39 responses were incomplete and/or incorrect. Subsequent outlier analysis excluded 36 participants. While analyzing the data, Shapiro–Wilk and Kolmogorov Smirnov tests were applied and the normality distribution of the data set was examined. The significance result obtained from the tests was *p* > 0.05 for the scales used in the research. The skewness and kurtosis values for the measurement tools were determined as (−0.80, 0.99) for Teacher Autonomy Scale, (−0.16, −0.03) for Collective Teacher Efficacy Scale, and (0.12, 0.31) for Positive Organizational Behavior Scale. With this result, it can be stated that the data meet the normal distribution conditions (Tabachnick and Fidell, 2013). Kaiser–Mayer–Olkin (KMO) coefficient and Bartlett’s test were performed to examine the suitability of the data for factor analysis. After testing the suitability of the scales for factor analysis, the theoretical model constructed as a result of the literature review was tested with structural equation modeling. In addition, Independent Sample *T*-Test, Pearson Correlation Test and descriptive statistics were utilized in the study.

## Results

In Table 2 the average score of the participants in the Teacher Autonomy Scale ( $\bar{x}$ = 4.14), Collective Efficacy Scale ( $\bar{x}$ = 3.62) and Positive Organizational Behavior Scale ( $\bar{x}$ = 3.88).

**Table 2:** Mean scores of participants’ teacher autonomy, collective efficacy and organizational behavior scales.

Scales	*N*	Min	Max	$\bar{x}$	SD
Teacher Autonomy	250	1.59	5.00	4.14	0.56
Collective Efficacy	250	2.08	5.00	3.62	0.54
Positive Organizational Behavior	250	2.29	5.00	3.88	0.47

In Table 3 it was concluded that participants’ teacher autonomy, collective efficacy and positive organizational behaviors did not differ according to gender variable, *t*_1_(248) = 0.32, *p* > 0.05, *t*_2_(248) = 0.71, *p* > 0.05, *t*_3_(248) = −0.91, *p* > 0.05.

**Table 3:** *T*-test results of mean scores of participants’ teacher autonomy, collective efficacy and positive organizational behavior scales according to gender variables.

Scales	Gender	*N*	$\bar{x}$	*S*	SD	*t*	*p*
Teacher Autonomy	Male	141	4.12	0.60			
	Female	109	3.65	0.54	248	0.71	0.48
Collective Efficacy	Male	141	3.60	0.55			
	Female	109	3.84	0.45	248	−0.91	0.36
Positive Organizational Behavior	Male	141	3.90	0.49			
	Female	109	4.15	0.52	248	0.32	0.75
	Total	250					

*p <* 0.05.

When Table 4 is considered, a positive and moderate relationship was found between the participants’ teacher autonomy and their collective efficacy (*r*_1_ = 0.32 *p* < 0.01). In addition, it was determined that there was a positive and moderate relationship between participants’ teacher autonomy and positive organizational behavior levels. With the determination of the relationships between the variables of the study, the predictive effect of teacher autonomy and collective efficacy on organizational behavior was determined by path analysis.

**Table 4:** Examination of the relationship between variables with Pearson product moment correlation.

Variable	Teacher Autonomy	Collective Efficacy	Positive Organizational Behavior
Teacher Autonomy	1		
Collective Efficacy	0.32**	1	
Positive Organizational Behavior	0.48**	0.39**	1

**p < 0.01.

**Figure 1:**
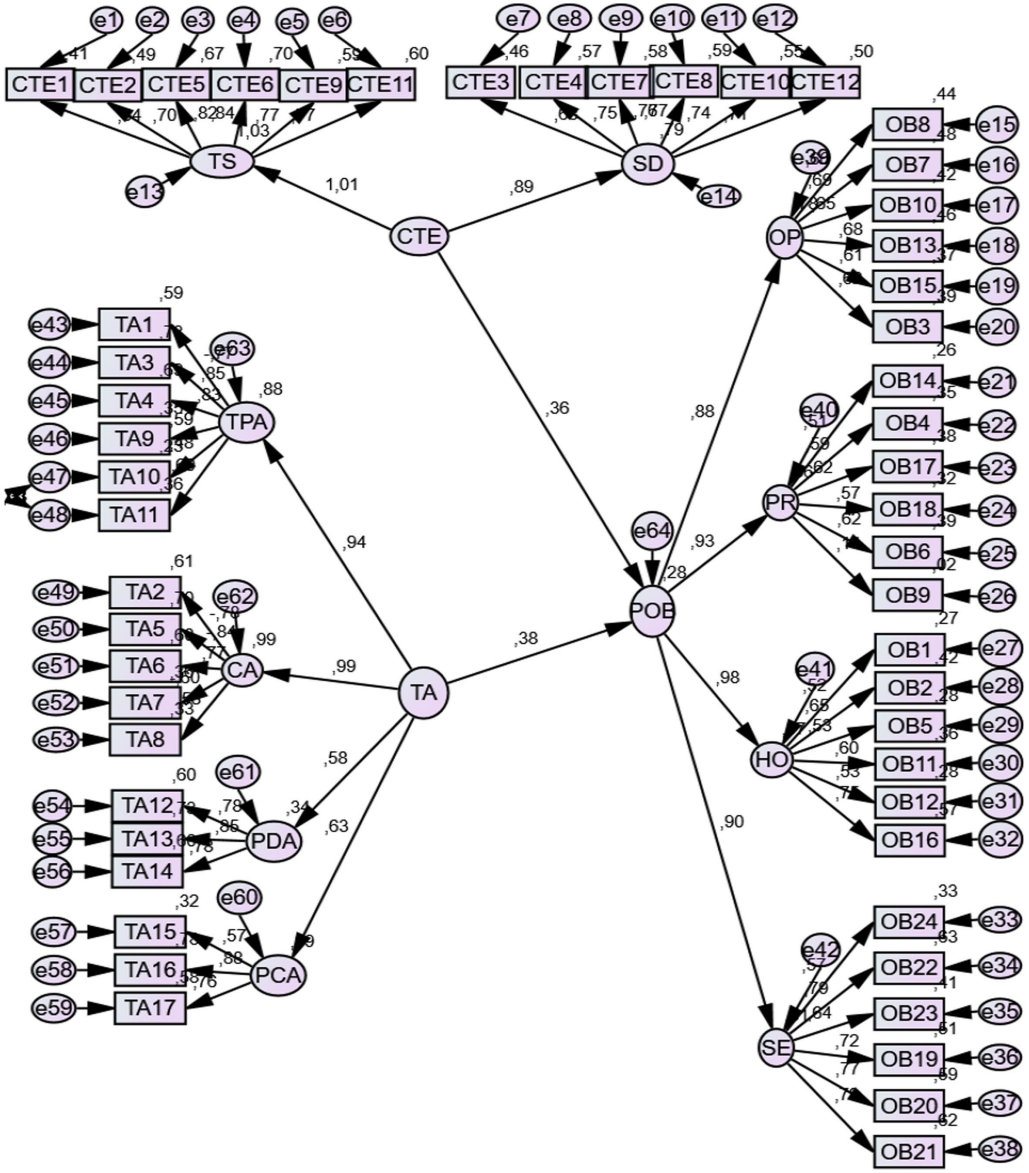
Structural equation model.

When the fit index values shown in Figure 1 are examined, it can be stated that the tested model appears to meet acceptable goodness-of-fit criteria based on commonly reported thresholds (*χ*^2^/df = 2.09, RMSEA = 0.06, PGFI = 0.65, PNFI = 0.66, GFI = 0.87, AGFI = 0.85, CFI = 0.91, CR = 79, AVE = 54). The analysis results are presented in Table 5.

**Table 5:** Structural equation model fit index values.

Model fit index	Excellent value range	Acceptable range	SEM
*χ*^2^/df	0 < *χ*^2^/df < 2	2 < *χ*^2^/df < 5	2.09
RMSEA	0.00 < RMSEA < 0.05	0.05 < RMSEA < 0.10	0.06
PGFI	0.95 < PGFI < 1.00	0.50 < PGFI < 0.95	0.65
PNFI	0.95 < PNFI < 1.00	0.50 < PNFI < 0.95	0.66
GFI	0.90 < GFI < 1.00	0.85 < GFI < 0.90	0.87
AGFI	0.90 < AGFI < 1.00	0.85 < AGFI < 0.90	0.85
CFI	0.95 < CFI < 1.00	0.90 < CFI < 0.95	0.91

*χ*^2^/df, chi-square divided by degrees of freedom; RMSEA, root mean square error of approximation; PGFI, parsimony goodness-of-fit index; PNFI, parsimony normed fit index; GFI, goodness-of-fit index; AGFI, adjusted goodness-of-fit index; CFI, comparative fit index.

After examining the goodness-of-fit index values for the model, the paths included in the model and the parameter estimates related to the model were analyzed. According to the constructed model, the standardized *β* coefficients, standard error, critical ratio, *p*, and *R*^2^ values between the variables are shown in Table 6.

**Table 6:** Structural equation model results.

Variables		Standardized *β*	Standard error	Critical ratio	*p*	*R* ^2^
Teacher Autonomy	Positive Organizational Behavior	0.35	0.07	5.27	***	0.28
Collective Efficacy		0.39	0.07	5.86	***	

***p < 0.001.

The results of the analysis indicate that there is a statistically significant effect of teacher autonomy and collective efficacy on positive organizational behavior (*β*_1_ = 0.35; *p* < 0.05; *β*_2_ = 0.39; *p* < 0.05). From this point of view, the findings provide statistical support for Hypotheses 1 and 2. However, teacher autonomy accounts for approximately 32% of the variance in collective efficacy, and collective efficacy explains 31% of positive organizational behavior. In addition, considering the findings of the model, it can be said that teacher autonomy and collective efficacy explain 28% of positive organizational behavior.

## Discussion

In the study, the effects of teacher autonomy and collective teacher efficacy of physical education and sport teachers on positive organizational behavior were determined. The first finding obtained in this direction is that the participants’ collective efficacy and positive organizational behavior levels are not at the targeted level, while their teacher autonomy levels are at a good level. It can be stated that the participants have the ability to decide on the teaching methods to be used in the lessons, to organize the subject content according to the needs of the students, to renew the teaching materials in the light of current information, and to apply appropriate assessment and evaluation methods. In addition, it can be stated that the participants think that they do not have a direct influence on their colleagues in terms of motivating students for academic success, making arrangements to support learning, understanding the course content in depth, contributing to the development of creativity skills, and supporting critical thinking skills.

It was determined that the collective efficacy of the participants did not show a significant difference according to the gender variable. When the relevant literature on collective efficacy is examined, it is possible to find studies that support the research results (Danış, 2020; Kapat, 2022; Klassen, 2010; Lin, 2013). In addition, there are also results that differ with the research results (Kurt, 2012; Zafer-Güneş, 2014). It is thought that the reason for the differences in the literature is that collective efficacy can be affected by culture, socioeconomic environment and educational level rather than gender differences. In addition, it was determined that teacher autonomy and positive organizational behavior did not differ according to gender variable. Considering the literature on teacher autonomy, it can be stated that there are studies that support the research results (Bayraktar, 2019; Çolak et al., 2017). However, it is also possible to mention studies with different results (Fadaee et al., 2021; Nasri et al., 2017; Ma et al., 2020). When positive organizational behavior and gender variable are examined, it is possible to mention studies that support (Barmola, 2013; Kurt, 2018) and do not support (Wilson, 2017) the research results. Therefore, when teacher autonomy and positive organizational behavior characteristics are taken into consideration, based on the current findings and the variability in the literature, it is difficult to draw a definitive conclusion regarding the role of gender. It is thought that the relevant result differences stem from interpersonal subjective factors and socio-demographic differences.

The finding related to the problem statement of the study is that teacher autonomy and collective efficacy have a significant effect on positive organizational behavior and have a 28% predictive power on positive organizational behavior. When the related literature is examined, it is possible to state that research on positive organizational behavior is handled from different perspectives (Jeung, 2011; Muse et al., 2008; Ross et al., 2012; Tindowen, 2019; Youssef and Luthans, 2007). In addition, it is also possible to come across studies in which teacher autonomy and collective efficacy are examined as independent variables (Budworth, 2011; DiLucchio and Leaman, 2022; Schnurr and Lohman, 2013; Sosic-Vasic et al., 2015; Marcionetti and Castelli, 2022). Buonomo et al. (2020) found that collective efficacy has a significant impact on job satisfaction, which is associated with positive organizational behavior. Furthermore, collective work can be said to be an element that enhances collaboration (Ganotice et al., 2022). Furthermore, collective efficacy has been found to be related to teacher self-efficacy (Choong and Ng, 2024), and teacher autonomy is known to be one of the factors that increase commitment in the workplace (Ahakwa, 2024). Kengatharan (2020) found that there was a positive relationship between teacher autonomy and teachers’ work behavior. Gülsün et al. (2023) state that teachers’ collective efficacy positively affects their behavior. However, no research examining the effect of collective efficacy and teacher efficacy on organizational behavior was found in the literature. In this case, it is anticipated that the findings may encourage further research on this topic.

## Conclusion

It can be stated that teachers’ characteristics such as being able to control some negative characteristics on students (cheating, preventing other students from learning, speaking without speaking, etc.), providing trust in the classroom environment, transferring a qualified educational content about the course subjects, being a figure of success, contributing to critical thinking skills, as well as characteristics such as in-class time management, organizing content according to student needs, applying correct communication with school administration, choosing in-service trainings and participating in scientific activities covering the field may influence their feelings, thoughts, attitudes, and behaviors within their institutions, during their tenure in their institutions. Therefore, enhancing the quality of teachers’ professional development and their familiarity with current scientific literature may contribute to positive behavioral changes at the institutional level. From this point of view, it is suggested that it may be beneficial to review teacher recruitment and employment policies in line with contemporary scientific criteria to enhance the overall quality of academic staff in the field.

### Strengths

The current study has several important strengths. The first of these is that some characteristics that shape the positive behaviors of physical education and sports teachers, who are seen as important figures in the field of sports and serve the field, have been determined. It has been determined that teacher autonomy and collective efficacy are important factors on positive organizational behavior and have a significant percentage of explanatory power. The study contributes to providing a working perspective to increase the quality of physical education and sports teachers.

In addition, the average score of the sample group on the variables discussed in the study and its meaning are described. In addition, the importance of gender on the variables is evaluated and the relevant results are presented. However, it can be seen in the relevant literature that the gender variable is subject to different results.

In the study, the use of structural equation modeling in the analysis of data is considered important in determining the direct and indirect effects on the variables considered, and in terms of the consistency of the explanatory power determined. Moreover, no research examining the effect of collective efficacy and teacher efficacy on organizational behavior was found in the literature. This aspect may indicate the potential originality and contribution of the study within the existing literature.

### Limitation

The sample of the study may not be representative of the general population, which may limit the generalizability of the findings. The study is limited by the measurement tools used. In addition, it is important to conduct more in-depth studies on these topics using qualitative research techniques (interviews, observation, etc.) or mixed research methods. The research is limited to a single city (Ankara). In addition, although a higher sample size was planned, the final number of participants constitutes another limitation of the research. Furthermore, the number of participants may have contributed to self-report bias, which is another limitation of the study.

## Data Availability

The raw data supporting the conclusions of this article will be made available by the authors, without undue reservation.

## References

[ref1] Ahakwa I. (2024). Enhancing teachers’ commitment: autonomy and learning in Ghana’s basic schools. Teach. Teach. Educ. 143:104556. doi: 10.1016/j.tate.2024.104556

[ref2] Amabile T. M. (1996). Creativity in Context: Update to the Componential Model of Creativity. Boulder, CO: Westview Press.

[ref3] Avolio B. J. Luthans F. (2006). Building Positive Organizations: A Strengths-Based Approach. New York, NY: Psychology Press.

[ref4] Bandura A. (1997). Self-Efficacy: The Exercise of Control. New York: W.H. Freeman.

[ref5] Barmola K. C. (2013). Gender and psychological capital of adolescents. Indian J. Appl. Res. 3, 1–3. doi: 10.15373/2249555X/OCT2013/142

[ref6] Bayraktar E. (2019). *The relationship between teachers’ perceptions of autonomy and their perceptions of organizational commitment*. (Master’s thesis). İstanbul Sabahattin Zaim University, Istanbul

[ref7] Budworth M. H. (2011). Individual learning and group performance: the role of collective efficacy. J. Workplace Learn. 23, 391–401. doi: 10.1108/13665621111154403

[ref8] Buonomo I. Fiorilli C. Benevene P. (2020). Unravelling teacher job satisfaction: the contribution of collective efficacy and emotions towards professional role. Int. J. Environ. Res. Public Health 17:736. doi: 10.3390/ijerph17030736, 31979251 PMC7037006

[ref9] Byrne B. M. (2013). Structural Equation Modeling with Mplus: Basic Concepts, Applications, and Programming. New York, NY: Routledge.

[ref10] Cameron K. S. Spreitzer G. M. (2012). The Oxford Handbook of Positive Organizational Scholarship. Oxford: Oxford University Press.

[ref11] Choong Y. O. Ng L. P. (2024). Shaping teachers’ organizational citizenship behavior through self-efficacy and trust in colleagues: moderating role of collective efficacy. BMC Psychol. 12:532. doi: 10.1186/s40359-024-02050-8, 39363327 PMC11451000

[ref12] Çolak İ. Altınkurt Y. (2017). The relationship between school climate and teachers’ autonomy behaviors. Educ. Adm. Theory Pract. 23, 33–71.

[ref13] Çolak İ. Altınkurt Y. Yılmaz K. (2017). The relationship between teachers’ autonomy behaviors and job satisfaction. Karadeniz Sos. Bilim. Derg. 9, 189–208.

[ref14] Danış M. (2020). *Teachers’ views on the relationship between collective teacher efficacy and structural empowerment of teachers*. (Master’s thesis). Bolu Abant İzzet Baysal University, Bolu

[ref15] Dansereau F. (1997). Theories of Group Behavior. Chicago, IL: University of Chicago Press.

[ref16] Deci E. L. Ryan R. M. (2000). The “what” and “why” of goal pursuits: human needs and the self-determination of behavior. Psychol. Inq. 11, 227–268. doi: 10.1207/S15327965PLI1104_01

[ref17] Diener E. Biswas-Diener R. (2008). Happiness: Unlocking the Mysteries of Psychological Wealth. Malden, MA: Blackwell Publishing.

[ref18] DiLucchio C. Leaman H. (2022). The impact of teacher research on classroom practice and teacher autonomy. Inq. Educ. 14:3. Available online at: https://digitalcommons.nl.edu/ie/vol14/iss2/3

[ref19] Erdoğan U. Dönmez B. (2015). Adaptation of the collective teacher efficacy scale to Turkish: a validity and reliability study. Educ. Adm. Theory Pract. 21, 345–366. doi: 10.14527/kuey.2015.013

[ref20] Fadaee E. Marzban A. Najafi Karimi S. (2021). Teacher autonomy and teaching styles: a gender-comparative study of Iranian EFL. Iran. J. Engl. Acad. Purp. 10, 1–14.

[ref21] Gagne M. Deci E. L. (2005). Self-determination theory and work motivation. J. Organ. Behav. 26, 331–362. doi: 10.1002/job.322

[ref22] Ganotice F. A. Jr. Chan L. Shen X. Lam A. H. Y. Wong G. H. Y. Liu R. K. W. . (2022). Team cohesiveness and collective efficacy explain outcomes in interprofessional education. BMC Med. Educ. 22:820. doi: 10.1186/s12909-022-03886-7 36447247 PMC9706965

[ref23] Gronn P. (2000). Understanding Groups: A Practical Guide. London: SAGE Publications.

[ref24] Gülsün I. Malinen O. P. Yada A. Savolainen H. (2023). Exploring the role of teachers’ attitudes towards inclusive education, their self-efficacy, and collective efficacy in behaviour management in teacher behaviour. Teach. Teach. Educ. 132:104228. doi: 10.1016/j.tate.2023.104228

[ref25] Hackman J. R. (1987). The Design of Work Teams. New York: John Wiley & Sons.

[ref26] Hackman J. R. Oldham G. R. (1976). Motivation Through the Design of Work: Test of a Theory. Ithaca, NY: Cornell University Press.

[ref27] Hoyle R. H. (1999). Structural Equation Modeling: Concepts, Issues, and Applications. Thousand Oaks, CA: SAGE Publications.

[ref28] Jeung C. W. (2011). The concept of employee engagement: a comprehensive review from a positive organizational behavior perspective. Perform. Improv. Q. 24, 49–69. doi: 10.1002/piq.20110

[ref29] Johnson D. W. Johnson R. T. (1989). Cooperation and Competition: Theory and Research. Edina, MN: Interaction Book Company.

[ref30] Kapat S. (2022). *Examining the effects of teachers’ collective efficacy behaviors on organizational culture*. (Master’s thesis). Gaziantep University, Gaziantep

[ref31] Karasar N. (2007). Scientific Research Method: Concepts, Principles, Techniques. Ankara: Nobel Yayin.

[ref32] Katzenbach J. R. Smith D. K. (1993). The Wisdom of Teams: Creating the High-Performance Organization. Boston, MA: Harvard Business School Press.

[ref33] Kengatharan N. (2020). The effects of teacher autonomy, student behavior and student engagement on teacher job satisfaction. Educ. Sci. Theory Pract. 20, 1–15. doi: 10.12738/jestp.2020.4.001

[ref34] Klassen R. M. (2010). Teacher stress: the mediating role of collective efficacy beliefs. J. Educ. Res. 103, 342–350. doi: 10.1080/00220670903383069

[ref35] Kramer R. M. Dutton J. E. (2001). The social identity perspective on organizational behavior. Res. Organ. Behav. 23, 287–323.

[ref36] Kurt T. (2012). Teachers’ perceptions of self-efficacy and collective efficacy. Turk. J. Educ. Sci. 10, 195–227.

[ref37] Kurt N. (2018). *The relationship between teachers’ psychological capital perceptions, psychological well-being and job satisfaction*. (Doctoral thesis). Gazi University, Ankara.

[ref38] Lin S. C. (2013). The relationships among teacher perceptions on professional learning community, collective efficacy, gender, and school level. J. Stud. Educ. 3, 98–111. doi: 10.5296/jse.v3i4.4387

[ref39] Locke E. A. Latham G. P. (1990). A Theory of Goal Setting and Task Performance. Englewood Cliffs, NJ: Prentice Hall.

[ref40] Luthans F. Avolio B. J. (2003). Authentic Leadership: Developing the Next Generation of Leaders. San Francisco, CA: Jossey-Bass.

[ref41] Luthans F. Avolio B. Avey J. Norman S. (2007). Positive psychological capital: measurement and relationship with performance and satisfaction. Pers. Psychol. 60, 541–572. doi: 10.1111/j.1744-6570.2007.00083.x

[ref42] Ma Y. Ma C. Lan X. (2020). Uncovering the moderating role of grit and gender in the association between teacher autonomy support and social competence among Chinese undergraduate students. Int. J. Environ. Res. Public Health 17:6398. doi: 10.3390/ijerph17176398, 32887420 PMC7504219

[ref43] Marcionetti J. Castelli L. (2022). Validation of a teacher self-efficacy scale in Italian and relations with relationship with col-leagues, school leadership, school ınnovativeness, teacher autonomy, role clarity, and role conflicts. Fla. Libr. 29, 281–295. doi: 10.4473/TPM29.3.1.

[ref44] Muse L. Harris S. G. Giles W. F. Feild H. S. (2008). Work-life benefits and positive organizational behavior: is there a connection? J. Organ. Behav. 29, 171–192. doi: 10.1002/job.506

[ref45] Nasri N. Vahid Dastjerdy H. Eslami Rasekh A. Amirian Z. (2017). Iranian EFL teachers’ practices and learner autonomy: do gender, educational degree, and experience matter? Innov. Lang. Learn. Teach. 11, 146–158. doi: 10.1080/17501229.2015.1078337

[ref46] Ross S. W. Romer N. Horner R. H. (2012). Teacher well-being and the implementation of school-wide positive behavior interventions and supports. J. Positive Behav. Interv. 14, 118–128. doi: 10.1177/1098300711413820

[ref47] Ryan R. M. Deci E. L. (2001). Self-determination theory and the facilitation of intrinsic motivation, social development, and well-being. Am. Psychol. 55, 68–78. doi: 10.1037/0003-066X.55.1.68, 11392867

[ref48] Ryan R. M. Deci E. L. (2002). “Overview of self-determination theory: an organismic dialectical perspective,” in Handbook of Self-Determination Research, (Rochester, NY: University of Rochester Press), 33–33.

[ref49] Ryan R. M. Deci E. L. (2008). “Self-determination theory and the role of basic psychological needs in personality and the organization of behavior,” in Handbook of Personality: Theory and Research, eds. John O. P. Robins R. W. Pervin L. A.. 3rd ed (New York, NY: The Guilford Press), 654–678.

[ref50] Salas E. Dickinson T. L. Converse S. A. Tannenbaum S. I. (2005). “Toward a model of team performance and training: insights from the aviation industry,” in Principles of Team Performance and Training, eds. Cannon-Bowers J. A. Smith-Jentsch S. J. (Mahwah, NJ: Lawrence Erlbaum Associates), 31–56.

[ref51] Schnurr M. P. Lohman B. J. (2013). The impact of collective efficacy on risks for adolescents’ perpetration of dating violence. J. Youth Adolesc. 42, 518–535. doi: 10.1007/s10964-013-9909-5, 23361319

[ref52] Sosic-Vasic Z. Keis O. Lau M. Spitzer M. (2015). The impact of motivation and teachers’ autonomy support on children’s executive functions. Front. Psychol. 6:146. doi: 10.3389/fpsyg.2015.00146, 25762958 PMC4327577

[ref53] Tabachnick B. G. Fidell L. S. (2013). Using Multivariate Statistics. 6th Edn Boston, MA: Pearson.

[ref54] Tindowen D. J. (2019). Influence of empowerment on teachers’ organizational behaviors. Eur. J. Educ. Res. 8, 617–631. doi: 10.12973/eu-jer.8.2.617

[ref55] Tschannen-Moran M. Barr M. (2004). Fostering student learning: the relationship of collective teacher efficacy and student achievement. Leadersh. Policy Sch. 3, 189–209. doi: 10.1080/15700760490503706

[ref56] Wageman R. (1995). Interdependence and group effectiveness. Admin. Sci. Q. 40, 145–180. doi: 10.2307/2393703

[ref57] West M. A. (2002). Effective Teamwork: Practical Lessons from Organizational Research. Malden, MA: Blackwell Publishing.

[ref58] Wilson F. M. (2017). Organizational Behaviour and Gender. London: Routledge.

[ref59] Youssef C. M. Luthans F. (2007). Positive organizational behavior in the workplace: the impact of hope, optimism, and resilience. J. Manage. 33, 774–800. doi: 10.1177/0149206307305562

[ref60] Zafer-Güneş D. (2014). *Examining the relationships between primary school teachers’ organizational trust and collective efficacy perceptions and their organizational awareness levels*. (Doctoral thesis). Abant Izzet Baysal University, Bolu

